# Supplementary material for: Determining the probability of hemiplasy in the presence of incomplete lineage sorting and introgression
Source: eLife. 2020 Dec 21;9:e63753. doi: 10.7554/eLife.63753 (PMC7800383; doi:10.7554/eLife.63753)
Supplement: Transparent reporting form [file elife-63753-transrepform.docx]

***eLife’s* transparent reporting form**

We encourage authors to provide detailed information *within their submission* to facilitate the interpretation and replication of experiments. Authors can upload supporting documentation to indicate the use of appropriate reporting guidelines for health-related research (see [EQUATOR Network](http://www.equator-network.org/%20)), life science research (see the [BioSharing Information Resource](https://biosharing.org/)), or the [ARRIVE guidelines](http://www.plosbiology.org/article/info:doi/10.1371/journal.pbio.1000412) for reporting work involving animal research. Where applicable, authors should refer to any relevant reporting standards documents in this form.

If you have any questions, please consult our Journal Policies and/or contact us: [editorial@elifesciences.org](mailto:editorial@elifesciences.org).

**Sample-size estimation**

- You should state whether an appropriate sample size was computed when the study was being designed
- You should state the statistical method of sample size computation and any required assumptions
- If no explicit power analysis was used, you should describe how you decided what sample (replicate) size (number) to use

Please outline where this information can be found within the submission (e.g., sections or figure legends), or explain why this information doesn’t apply to your submission:

We did not conduct a traditional power analysis as we did not perform any experiments in our study. The number of replicate simulations to perform in our benchmarking analyses and empirical case studies was determined via guidelines available on the README of the GitHub repository for our software *HeIST*.

**Replicates**

- You should report how often each experiment was performed
- You should include a definition of biological versus technical replication
- The data obtained should be provided and sufficient information should be provided to indicate the number of independent biological and/or technical replicates
- If you encountered any outliers, you should describe how these were handled
- Criteria for exclusion/inclusion of data should be clearly stated
- High-throughput sequence data should be uploaded before submission, with a private link for reviewers provided (these are available from both GEO and ArrayExpress)

Please outline where this information can be found within the submission (e.g., sections or figure legends), or explain why this information doesn’t apply to your submission:

The number of replicate simulations performed for each benchmarking condition and empirical use case is reported in the Materials & Methods section. We did not perform any experiments, so there is no discussion of biological vs. technical replicates or outliers.

**Statistical reporting**

- Statistical analysis methods should be described and justified
- Raw data should be presented in figures whenever informative to do so (typically when N per group is less than 10)
- For each experiment, you should identify the statistical tests used, exact values of N, definitions of center, methods of multiple test correction, and dispersion and precision measures (e.g., mean, median, SD, SEM, confidence intervals; and, for the major substantive results, a measure of effect size (e.g., Pearson's r, Cohen's d)
- Report exact p-values wherever possible alongside the summary statistics and 95% confidence intervals. These should be reported for all key questions and not only when the p-value is less than 0.05.

Please outline where this information can be found within the submission (e.g., sections or figure legends), or explain why this information doesn’t apply to your submission:

Reporting of our statistical analyses of can be found in the Materials and Methods under “Accuracy of *HeIST*” and “Empirical applications of *HeIST*”; in the Results under “*HeIST* effectively captures the effects of ILS and introgression on hemiplasy risk”, “The distribution of geeen-blooded New Guinea lizards is likely to have arisen from fewer than four transitions” and “A chromosomal inversion in the *Heliconius erato/sara* clade likely has a single origin”; in Figures 1, 5, and 6; in the Figure Supplements; and in the tables in Supplementary File 1.

(For large datasets, or papers with a very large number of statistical tests, you may upload a single table file with tests, Ns, etc., with reference to sections in the manuscript.)

**Group allocation**

- Indicate how samples were allocated into experimental groups (in the case of clinical studies, please specify allocation to treatment method); if randomization was used, please also state if restricted randomization was applied
- Indicate if masking was used during group allocation, data collection and/or data analysis

Please outline where this information can be found within the submission (e.g., sections or figure legends), or explain why this information doesn’t apply to your submission:

Not applicable – we did not perform any experiments or generate any new data sets.

**Additional data files (“source data”)**

- We encourage you to upload relevant additional data files, such as numerical data that are represented as a graph in a figure, or as a summary table
- Where provided, these should be in the most useful format, and they can be uploaded as “Source data” files linked to a main figure or table
- Include model definition files including the full list of parameters used
- Include code used for data analysis (e.g., R, MatLab)
- Avoid stating that data files are “available upon request”

Please indicate the figures or tables for which source data files have been provided:

Availability of the lizard genomic data and *Heliconius* phylogenetic network is detailed in the Acknowledgements section of the source manuscript. Source code and test cases for our software *HeIST* are freely available from the GitHub repository. Source data files have been provided for Figures 1, 4, 5, and 6 of the main text. The Appendix details all the mutation rate parameters of our mathematical model.
